# Investigation of the *Plasmodium falciparum* Food Vacuole through Inducible Expression of the Chloroquine Resistance Transporter (PfCRT)

**DOI:** 10.1371/journal.pone.0038781

**Published:** 2012-06-13

**Authors:** Florian Ehlgen, James S. Pham, Tania de Koning-Ward, Alan F. Cowman, Stuart A. Ralph

**Affiliations:** 1 Department of Biochemistry and Molecular Biology, Bio21 Molecular Science and Biotechnology Institute, The University of Melbourne, Parkville, Victoria, Australia; 2 School of Medicine, Deakin University, Waurn Ponds, Victoria, Australia; 3 Division of Infection and Immunity, The Walter and Eliza Hall Institute of Medical Research, Parkville, Victoria, Australia; Université Pierre et Marie Curie, France

## Abstract

Haemoglobin degradation during the erythrocytic life stages is the major function of the food vacuole (FV) of *Plasmodium falciparum* and the target of several anti-malarial drugs that interfere with this metabolic pathway, killing the parasite. Two multi-spanning food vacuole membrane proteins are known, the multidrug resistance protein 1 (PfMDR1) and Chloroquine Resistance Transporter (PfCRT). Both modulate resistance to drugs that act in the food vacuole. To investigate the formation and behaviour of the food vacuole membrane we have generated inducible GFP fusions of chloroquine sensitive and resistant forms of the PfCRT protein. The inducible expression system allowed us to follow newly-induced fusion proteins, and corroborated a previous report of a direct trafficking route from the ER/Golgi to the food vacuole membrane. These parasites also allowed the definition of a food vacuole compartment in ring stage parasites well before haemozoin crystals were apparent, as well as the elucidation of secondary PfCRT-labelled compartments adjacent to the food vacuole in late stage parasites. We demonstrated that in addition to previously demonstrated Brefeldin A sensitivity, the trafficking of PfCRT is disrupted by Dynasore, a non competitive inhibitor of dynamin-mediated vesicle formation. Chloroquine sensitivity was not altered in parasites over-expressing chloroquine resistant or sensitive forms of the PfCRT fused to GFP, suggesting that the PfCRT does not mediate chloroquine transport as a GFP fusion protein.

## Introduction

The parasite *Plasmodium falciparum* is the causative agent of severe malaria in humans, killing somewhere between 600,000 and 1.2 million people each year [1], [2]. The complex life cycle includes an asexual proliferation within human erythrocytes, characterised by three distinct stages: rings, trophozoites and schizonts. Adaptations to the intra-erythrocytic lifestyle have created new and in some cases unique organelles, such as an endosomal/lysosomal-like organelle, the food vacuole (FV) (reviewed in [3]).

In contrast to other organelles, such as the mitochondrion and the apicoplast [4], the FV does not persist throughout the whole asexual intra-erythrocytic life cycle, and is discarded at the end of each cycle. Analysis by multiple laboratories has resulted in several competing models for FV biogenesis during the ring stage. These include 1) The “big gulp model”, which suggests that the first step in the FV biogenesis is an initial uptake of a single large parcel of haemoglobin followed by a continuous uptake of packages mediated by cytostomes that fuse, vesicle-like, with the FV. An additional contribution of endocytosis is not excluded from this model [5]. 2) The “tubus model” prefers the contribution of cytostomes in the uptake of haemoglobin as well, yet in contrast to the previous model this theory is vesicle-independent. In this model, the cytostomes do not initially pinch-off but form elongated tubes that eventually fuse with the FV. The tubes would initially enable the uptake of haemoglobin through contact with the cytostome, and then later deliver haemoglobin to the FV after pinching off and fusing with the FV [6]. 3) The “pre-FV model” is based on the observation that in early intra-erythrocytic life stages multiple enclosed compartments, called pre-FV compartments, are detectable in which haemoglobin degradation has already started [7]. In late ring stages these smaller compartments fuse and give rise to the FV in early trophozoite stages. Vesicles derived from the cytostomal invagination are delivered to the FV to continue supplying the organelle with haemoglobin [7].

The degradation of haemoglobin is still the only verified function of the FV, and has been studied extensively [3]. The protein component of haemoglobin is catabolised via series of peptidases [8], [9], [10] but this degradation releases ferriprotoporphyrin IX (FP) which cannot be degraded by the malarial parasite and is toxic. Instead, *Plasmodium* parasites polymerise FP to crystalline haemozoin that becomes a distinct feature of the FV of trophozoite and schizont parasites [11], [12], [13]. The crucial process of FP polymerisation is the target of important antimalarial drugs. The best understood of these inhibitory processes is that of chloroquine (CQ), which accumulates in the FV and there inhibits the polymerisation of haem into haemozoin, either by binding free FP [14], or by capping FP polymers [15]. This results in accumulation of toxic unpolymerised FP and toxic FP-CQ complexes, although there is ongoing discussion of exactly how these compounds mediate parasite death [16], [17].

Chloroquine enters parasites in a neutral form that is believed to diffuse across the food vacuole membrane, but inside the acidic environment it is protonated and, once charged, is unable to freely diffuse out of the FV again. This leads to an accumulation of chloroquine inside the food vacuole, relative to the rest of the parasite and the external milieu. Chloroquine resistant parasites accumulate considerably less CQ in their FV relative to sensitive parasites [18], [19], [20], [21]. The difference in accumulation is attributable to mutations in the FV membrane protein PfCRT (PF3D7_0709000), particularly a substitution of K to T at residue 76 [22] and to a lesser degree, changes in the FV membrane protein PfMDR1 (PF3D7_0523000) [23], [24]. The means by which PfCRT modulates FV chloroquine concentration has been much debated, and some authors have suggested that a difference in the pH of the FV between sensitive and resistant parasites modified the accumulation of drug (eg [25]). However, more recent studies indicate that sensitive and resistant parasites do not have significantly different FV acidity [26], but that the modified PfCRT in CQ resistant parasites directly facilitates a leak of chloroquine out of the FV [27], [28], [29], [30]. These data indicate that mutations in PfCRT allow charged CQ to escape from the FV, and that its efflux decreases the concentration of CQ in the FV to non-lethal levels. Despite its clear association with resistance, it remains unclear whether PfCRT mutations are sufficient to generate chloroquine resistance in the field. In many chloroquine resistant isolates, polymorphisms are also found in PfMDR1 and other transporters [31], and regulatory changes in assorted other genes have been associated with compensation for the resistance mutations in PfCRT [32]. Even in isogenic lines, transfected parasites take several weeks to reach population levels that allow drug assays to be performed, potentially allowing lines to compensate for PfCRT changes through secondary *trans* mutations or epigenetic regulatory changes. To circumvent the necessarily long delay between transfection and analysis of transgenic parasites, and to characterise the direct effect of PfCRT mutations, we generated isogenic parasites lines with anhydrotetracycline-regulatable expression of PfCRT-GFP fusions, using *pfcrt* alleles from sensitive (PfCRT^S^) and resistant parasites (PfCRT^R^).

Inducible expression of PfCRT-GFP fusions also allows us to further characterise the trafficking of PfCRT to the FV membrane. Our knowledge about the targeting of proteins towards the FV has until recently been restricted to lumenal proteases participating in haemoglobin degradation, such as Plasmepsin II and DPAP1. The FV lumenal peptidase Plasmepsin II is targeted via the parasite plasma membrane (PM) to the FV as an integral membrane protein in a Brefeldin A (BFA)-sensitive manner [8], whereas the soluble DPAP1 is first transported through the PM, accumulating in the Parasitophorous vacuole (PV), followed by the final transport step to the FV [8]. Recently the first study of targeting for PfCRT was published, demonstrating that PfCRT traffic to the FV membrane is via the ER, is Brefeldin A sensitive, and that subsequent direction to the FVM depends on a small region at the C-terminus that includes a potentially phosphorylated threonine (position 416) [33]. The key PfCRT mutation associated with chloroquine resistance, K76T, is positioned at the boundary of the protein's first transmembrane domain (TMD), and the length and composition of a protein's initial transmembrane length is a trafficking determinant in other endomembrane proteins [34], [35], [36]. We therefore examined whether the change of charge at the transmembrane boundary of PfCRT affected the sub-cellular localisation and thus function of the CQ-resistant form.

In this study we have taken advantage of an inducible expression system in order to over-express both PfCRT^S^ and PfCRT^R^ as GFP-tagged fusion proteins within CQ sensitive 3D7 *Plasmodium* parasites. We show that both PfCRT forms are identically localised at the FV membrane, and that differential subcellular localisation is therefore not a cause of differential chloroquine sensitivity. We also show that PfCRT trafficking is inhibited by Dynasore, an inhibitor of dynamin-mediated vesicle formation, consistent with a role for dynamin in FV biogenesis. The inducible GFP-tagged PfCRT also provides an excellent tool to follow the biogenesis of the FV development. We show the existence of a PfCRT enveloped organelle prior to the appearance of haemozoin, the traditional definition of the appearance of FV.

## Materials and Methods

### Bioinformatic analysis

Sequence of the annotated PfCRT (PF3D7_0709000) was acquired from PlasmoDB [37] (www.plasmodb.org). Characteristic point mutations within the first TMD of PfCRT of the CQ-sensitive strain D10 and CQ-resistant strains 7G8 and Dd2 were obtained from Fidock et al. [22]. Alignments were performed using ClustalW (by Jalview [38]). Prediction of TMD length and position of PfCRT^S^ or PfCRT^R^ was carried out using the predictors TMHMM2 [39] (www.cbs.dtu.dk/services/TMHMM), MEMSAT [40] (http://saier-144-21.ucsd.edu/barmemsat.html) and SPLIT4 [41] (http://split.pmfst.hr/split/4/). Sequence of the first TMD of the glycoprotein M1 of the avian coronavirus infectious bronchitis virus was obtained from Machamer et al. [42]. Helical wheel projections were performed using the helical wheel applet created by Edward K. O'Neil and Charles M. Grisham (University of Virginia in Charlottesville, Virginia, USA).

### Plasmid construction

Plasmid pT150HA was generated by replacing the MSP2 5′UTR in pTMSP2HA that drives transcription of the TATi3 transactivator (a derivative of pTGFP that contains a dual haemagglutinin epitpope in place of GFP [43], [44] with that of PTEX150 (PF3D7_1436300). For this, *P. falciparum* gDNA was PCR amplified with the oligonucleotides PF0344-5′F (cggcttaagGTTGTTTTTCTCTTTG-TGGTCAAAATAAG) and PF0344-5′R (gctaatattattattctcatctcgagTTTTTTTTTTT-TTTTAAATGTTGAATTATAAAC), and the resulting 1.55 kb product was digested with *Afl*II and *Xho*I and then filled in with Klenow. This fragment was subsequently cloned into the *Xma*I/*Bst*B1 sites of pTMSP2HA that had also been filled in with Klenow. The 2xHA epitope tag of pT150HA was then replaced with GFP to create pT150GFP. This vector provides unique *Pst*I and *Mlu*I restriction enzyme sites between the minimal regulatable promoter and GFP to create inducible GFP fusion proteins. To create pT150KPfCRTGFP, sequences corresponding to the full-length coding region of PfCRT (PF3D7_0709000) were amplified from the chloroquine sensitive strain 3D7 (hereafter referred to as PfCRT^S^) and the chloroquine resistant 7G8 (hereafter referred to as PfCRT^R^), and directionally cloned into the *Pst*I and *Mlu*I site of pT150GFP.

### Parasite culture, transfection and induction of expression

O+ human erythrocytes (Australian Red Cross Blood Service, Melbourne, Australia) infected with *P. falciparum* parasites of the 3D7 or W2mef strain were cultured using a modification of the method established by Trager and Jensen [45] as described in [46]. Transfection of *P. falciparum* parasites was carried out as previously described [47]. A total of 100 µg plasmid-DNA was used per transfection and electroporated into ring-stage parasites.

*P. falciparum* parasites transfected with the inducible vector were recovered in HEPES-buffered RPMI-1640 medium supplemented with 3.6% sodium bicarbonate and 5% Albumax II (Invitrogen-Gibco) containing the transcription inhibitor anhydrotetracycline (ATc) (Sigma-Aldrich) at a final concentration of 0.5 µg/mL. WR99210 was added 4–5 h post transfection to the complete medium to a final concentration of 10 µM to select for parasites with the plasmid. The cultures were incubated in an atmosphere of 5% CO_2_, 1% O_2_, and 94% N_2_ at 37°C.

Protein expression from the ATc-repressible plasmid was induced as follows: Parasite cultures were synchronised using 5% sorbitol for 5 min at 37°C [48], and subsequently cultured in the absence of the transcription inhibitor ATc for 48 h. Following this, the parasitaemia was adjusted to 4–8% ring-stage parasites and incubated for further 18–24 h. Protein expression of GFP-epitope tagged proteins was checked using a Zeiss Axioplan 2 imaging Universal Microscope (Zeiss). Parasites in which protein expression had been induced were discarded after use, and subsequent experiments were performed with freshly induced parasites from a population of never-induced parasites.

### Western blot detection of overexpressed protein

Protein expression of GFP-tagged PfCRT was induced and total protein from erythrocytes infected with mainly schizont parasites was obtained by lysis with PBS containing 0.15% (w/v) saponin and 0.1% (w/v) BSA for 10 min at 4°C in the presence of EDTA-free protease inhibitor (Roche). Proteins from 1×10^7^ parasites were heated for 30 min at 40°C in the presence of EDTA-free protease inhibitor (Roche) and separated on a 10% Bis-Tris gel with MOPS running buffer (Invitrogen). Proteins were transferred to Hybond polyvinylidene fluoride (PVDF) membrane (GE Healthcare Life Sciences). GFP-tagged PfCRT was identified with mouse anti-GFP (1∶500, Roche) followed by anti-mouse HRP-conjugated IgG (1∶5,000, Molecular Probes), while endogenous GAPDH was identified with rabbit anti-GAPDH (1∶5,000) (A kind gift from Leann Tilley, University of Melbourne) followed by anti-rabbit HRP-conjugated IgG (1∶5,000, Molecular Probes) and visualised using chemiluminescence system (ECL, Pierce).

### Live cell imaging and immunofluorescence assays

Sample preparation of living cells was performed as follows: Protein expression of GFP-tagged PfCRT was induced, erythrocytes infected with mainly trophozoite and schizont parasites were harvested and the nuclei of the parasites stained with DAPI Nucleic Acid stain (Invitrogen) (1∶500) in PBS for 15 min at 4°C. Cells were washed once with PBS and resuspended in 12 µL PBS.

Immunofluorescence assays (IFAs) were performed as previously described [49]. Protein expression of GFP-tagged PfCRT was induced and erythrocytes infected with mainly trophozoite and schizont parasites were fixed for 50 min at room temperature (RT) in PBS containing 4% (v/v) paraformaldehyde and 0.0075% (v/v) glutaraldehyde, washed twice with ice-cold PBS, permeabilised with 0.01% Triton X-100 in PBS for 10 min at RT and washed with PBS. Cells were incubated with mouse anti-GFP (1∶200, Roche) antibody in 3% (w/v) BSA in PBS overnight at 4°C. Cells were washed twice with and incubated with either Alexa Fluor 488- or Alexa Fluor 594-conjugated antibodies (1∶500, Molecular probes) in 3% (w/v) BSA in PBS for 1 h at 4°C. Washed cells were incubated with DAPI Nucleic Acid stain (1∶500, Invitrogen) in 3% (w/v) BSA in PBS for 15 min at 4°C. Before mounting cells on a glass slide, they were washed twice in PBS and eventually resuspended in 12 µL PBS. A 5 µL aliquot of the resuspended cells was mounted in 60% glycerol with DABCO (Sigma-Aldrich) on a glass slide, covered with a cover slip coated with 0.01% or 0.1% Polyethylenimine for living cells or fixed cells, respectively, and sealed. The cells were analysed using the Zeiss Axioplan 2 imaging Universal Microscope (Zeiss) and the imaging software AxioVision v4.5 (Zeiss).

### Brefeldin A and Dynasore treatment

Erythrocytes infected with *P. falciparum* parasites were treated with either Brefeldin A (Sigma-Aldrich) as previously described [8] or Dynasore hydrate (Sigma-Aldrich) as previously described [50].

Brefeldin A (5 mg/mL dissolved in 100% ethanol) was added to young trophozoite or schizont parasites expressing GFP-tagged PfCRT following induction of protein expression to a final concentration of 5 µg/mL and incubated for 3 h. Live cell imaging or an IFA was performed as previously described. As a negative control, a second parasite culture was prepared in the same way, treating with the same volume of ethanol instead of Brefeldin A.

Protein expression of GFP-tagged PfCRT was induced and young trophozoite or schizont parasites were treated with 40 µmol/L Dynasore (200 mM dissolved in 100% (w/v) DMSO) for 2 h. Live cell imaging or an IFA was performed as previously described. A second parasite culture was prepared in the same way, however instead of Dynasore hydrate the same volume of 100% (v/v) DMSO was added.

### Chloroquine drug sensitivity assays

The drug sensitivity assays were performed using the SYBR Green staining method after 48 hours of growth as previously described by Smilkstein *et al*. [51] with some modifications as detailed in [52].

Protein expression of sensitive and resistant form of PfCRT as GFP-fusion protein in 3D7 *P. falciparum* parasites was induced as described above. A second corresponding parasite population was treated equally, yet maintained in the presence of the transcription inhibitor ATc. Wild type 3D7 and W2mef parasites were also assayed. Parasites of each population were dispensed to a final parasitaemia of 0.5% in triplicate to a 96-well tissue culture plate prepared with varying CQ concentration, 2% haematocrit, 10 µM selective agent WR99210 and in the case of uninduced parasites, 0.5 µg/mL ATc in a total volume of 200 µL. For relevant experiments, parasites were also cultured in 5 µM verapamil. Parasite growth of induced and uninduced parasites was also measured in the absence of CQ (positive control), and uninfected erythrocytes cultured in the absence of CQ, WR99210 and ATc provided the background level of fluorescence (negative control). Parasites were cultured for 48 h under optimal culturing conditions. Following the 48 h incubation period, CQ-treated parasites were washed, added to a new 96-well tissue culture plate containing SYBR Green I (Invitrogen) in lysis buffer (20 mM Tris pH 7.5, 5 mM EDTA, 0.008% (w/v) saponin, 0.08% (w/v) Triton X-100) and incubated for 1 h at RT. Fluorescence, with excitation and emission wavelength centred at 485 nm and 530 nm respectively, was examined using a ThermoScientific Varioskan Flash Microplate Reader. Experiments were conducted in triplicate, and each such experiment was performed independently five times.

## Results

### Expression of PfCRT^S^ and PfCRT^R^ as GFP-fusion protein

**Figure 1:**
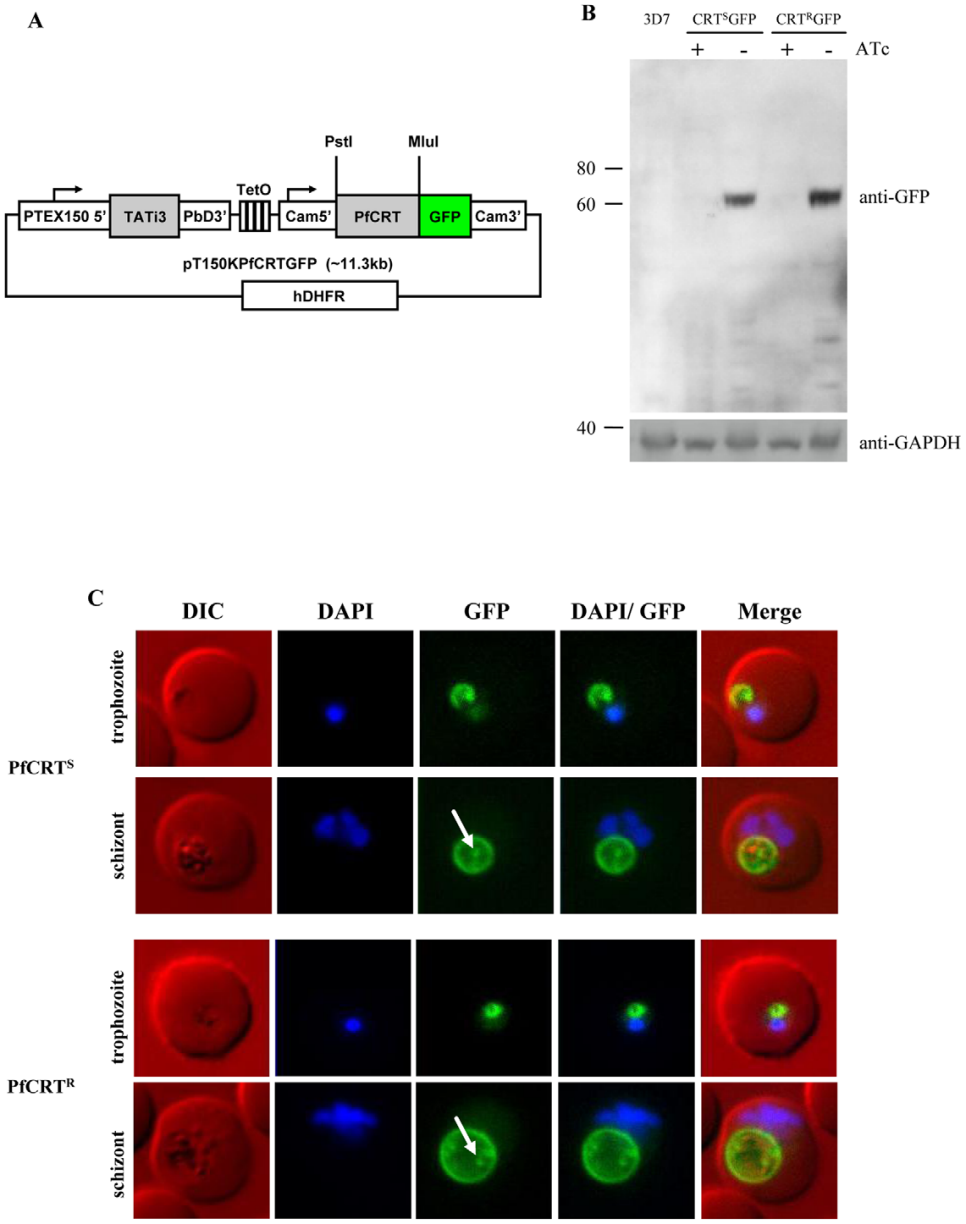
Over-expression of sensitive and resistant form of PfCRT as GFP-fusion protein. (**A**) Schematic representation of the inducible expression plasmid pT150KPfCRTGFP. Expression of the transcription activator TATi3 is under the control of the 5' UTR of PTEX150. In the presence of the transcription inhibitor anhydrotetracycline (ATc), TATi3-binding to the Tet operon (TetO) is inhibited and no expression of GFP-tagged PfCRT occurs. In the absence of ATc, TATi3 binds to TetO, initiating the over-expression of PfCRT-GFP. Cam5': 5' UTR of Calmodulin, hDHFR: human dihydrofolate reductase. (**B**) Western Blot analysis of induced over-expression of sensitive (PfCRT^S^) and resistant (PfCRT^R^) form of PfCRT as GFP-fusion. Presence of PfCRT^S^ and PfCRT^R^ as GFP-fusions, labelled with mouse anti-GFP, is confirmed by the presence of a band at 62 kDa only in the parasites cultured in the absence of ATc. *P. falciparum* wild-type strain 3D7 represents the negative control. Labelling with rabbit anti-GAPDH shows equal loading in these lanes. (**C**) Fluorescence microscopy of GFP-fusions of sensitive (PfCRT^S^, top) and resistant (PfCRT^R^, bottom) form of PfCRT. PfCRT^S^ and PfCRT^R^ were over-expressed as GFP-fusion proteins using an ATc-inducible expression system. Live cell images of DAPI-stained infected red blood cells show that both forms of PfCRT localise to the FV membrane. Later stages of the asexual life cycle show ring/dot-like structures (white arrow) within the FV, possibly degraded GFP-fusion proteins.

To investigate the phenotype of chloroquine sensitive parasites expressing a resistant form of PfCRT without a delay that would allow cryptic compensatory mutations and epigenetic changes [32], we generated ATc-regulatable constructs with PfCRT fused to GFP. We took advantage of the TATi3 transactivator system established by Meissner and colleagues that allows repression of expression by addition of ATc [44]. The original ATc-regulatable plasmid expressed the transactivator under the control of the promoter of PfMSP2 (PF3D7_0206800) [44], a gene with a temporal transcription peak in schizont stage parasites. To mimic the temporal expression of PfCRT we modified the TATI3 vector by replacing the PfMSP2 with the promoter of PfPTEX150 (PF3D7_1436300), a gene with very similar expression phase and abundance to PfCRT [53], [54]. GFP fusions were generated for the full gene of PfCRT from the chloroquine sensitive line 3D7 and the chloroquine resistant line 7G8, which contains, among other mutations, the K76T replacement (Figure 1A). Western blot analysis using an anti-GFP antibody against mixed parasite showed a band at a height corresponding to motility of ∼62 kDa products in the induced parasites, but not in uninduced samples (Figure 1B). 62 kDa is slightly smaller than the expected molecular weight of 76 kDa for PfCRT as a GFP fusion but multi-spanning TM proteins regularly migrate more quickly than expected from their apparent mass, and PfCRT alone was previously reported to migrate faster than expected [22]. These analyses validated this expression system for the inducible production and localisation of PfCRT^S^ and PfCRT^R^ (Figure 1C).

### Localisation of GFP-tagged PfCRT^S^ and PfCRT^R^ to the FV membrane

**Figure 2:**
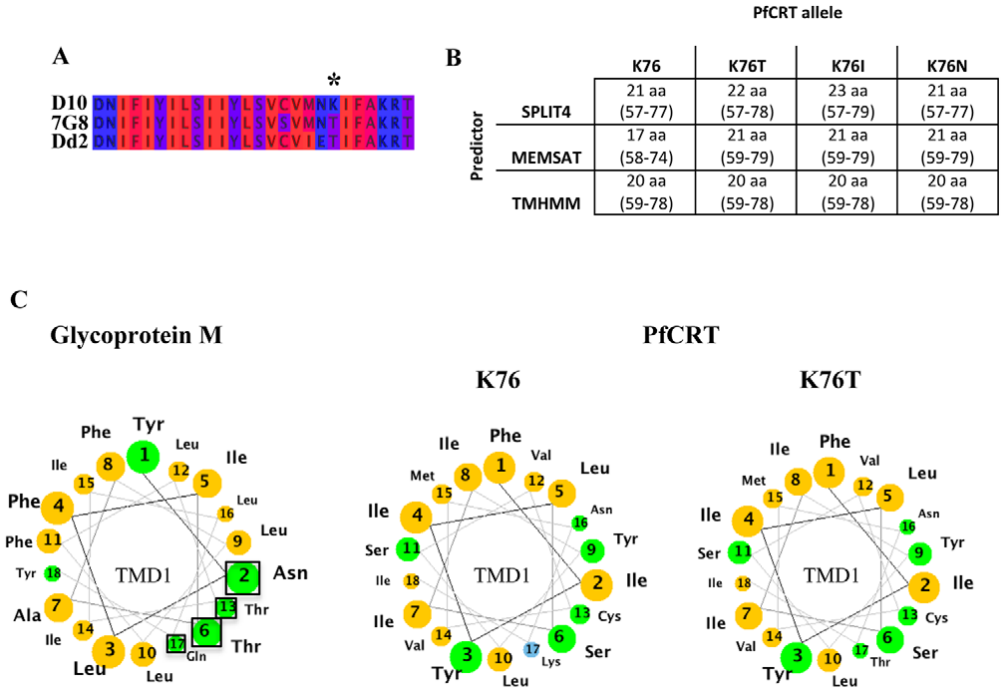
Bioinformatic analysis of the K76T mutation in PfCRT. (**A**) Multiple sequence alignment (by Jalview [38]) of the first TMD of CQ-sensitive D10 and CQ-resistant 7G8 and Dd2 parasites [22]. The amino acids are represented according to their hydrophobicity (hydrophilic residue (blue), hydrophobic residue (red), intermediate stages (dark red, violet)). The mutation at position 76 represents a replacement of a positively charged, hydrophilic residue (lysine, K) with an uncharged, hydrophobic (threonine, T) residue. (**B**) Predicted length of the first TMD of sensitive and various resistant alleles of PfCRT using different bioinformatic predictors. Single point mutations at position 76 [22], [63] were analysed using three different TM prediction applications, i.e. TMHMM2 [39], Memsat [40] and Split4 [41]. Following the TM prediction the predicted length and position of the first TMD of PfCRT were extracted and analysed. In contrast to TMHMM2, the predictors Memsat and Split predict a change in both length and position due to a single point mutation at position 76. (**C**) Helical wheel projection of the first TMD of Glycoprotein M (**left**) and PfCRT (**right**). Yellow residues are nonpolar; green residues are polar, uncharged; blue residues are basic. The first TMD of the infectious bronchitis virus Glycoprotein M consists of a polar face (black square around the involved residues) within its first TMD that becomes obvious when represented in a helical wheel projection (modified from [42]). Point mutations that modified the polar face of the TMD resulted in a mistargeting of the viral Glycoprotein M [42]. Helical wheel projection of the first TMD of PfCRT reveals a change in the polar face as a result of mutations at position 76 (modified from [62]).

Previous localisation studies of PfCRT^S^ have reported localisation solely to the FV membrane [22], [33]. However, our bioinformatic analysis of PfCRT^S^ and PfCRT^R^ predicted a possible difference in the length of the first TMD between resistant and sensitive proteins, associated with the mutations at position 76. Two independent TMD predictors, MEMSAT [40] and SPLIT4 [41], predicted that the mutations found in the PfCRT proteins of the resistant parasites DD2 or 7G8 (particularly changes at k76) would change the length, position and polar face of the first transmembrane domain (Figure 2A, B, C). Even small differences in TMD composition or length can be enough to mistarget proteins in the early stages of the secretory pathway [34], [35], [42], [55]. We therefore investigated whether the K76 mutations in PfCRT altered trafficking to the FV, which may impact its role in modulating drug resistance.

Live cell fluorescence as well as immunofluorescence assays were performed using the induced PfCRT^S^-GFP fusion protein. These localisation studies confirmed the localisation of PfCRT^S^ to the FV membrane (Figure 1C). Occasionally, parasites showed a minor additional fraction at the nuclear periphery, corresponding to patterns previously described for ER localisation [33]. The fluorescence signal remained strong over the whole intra-erythrocytic life stages. PfCRT-GFP in schizont stage parasites additionally localised to mobile puncta within the FV, as well as labelling at the FV membrane. This may represent internalised vesicles carrying PfCRT-GFP, or GFP alone after degradation of the fusion protein, although we did not see a GFP-only band by western blot.

The localisations of both PfCRT^S^ and PfCRT^R^ were simultaneously analysed and compared repeatedly, but no differences in the localisation were observed between the two at any life stage (Figure 1). Both forms show a clear and restricted localisation to the FV membrane, including the particularities mentioned above, such as occasional ER staining and puncta within the FV lumen in older parasites. These results confirmed that the inducible expression system used in this study was capable of expressing both forms of PfCRT as GFP-fusions and that the K76T mutation does not result in any altered localisation of PfCRT.

### Development of the FV during the intra-erythrocyte life stages

**Figure 3:**
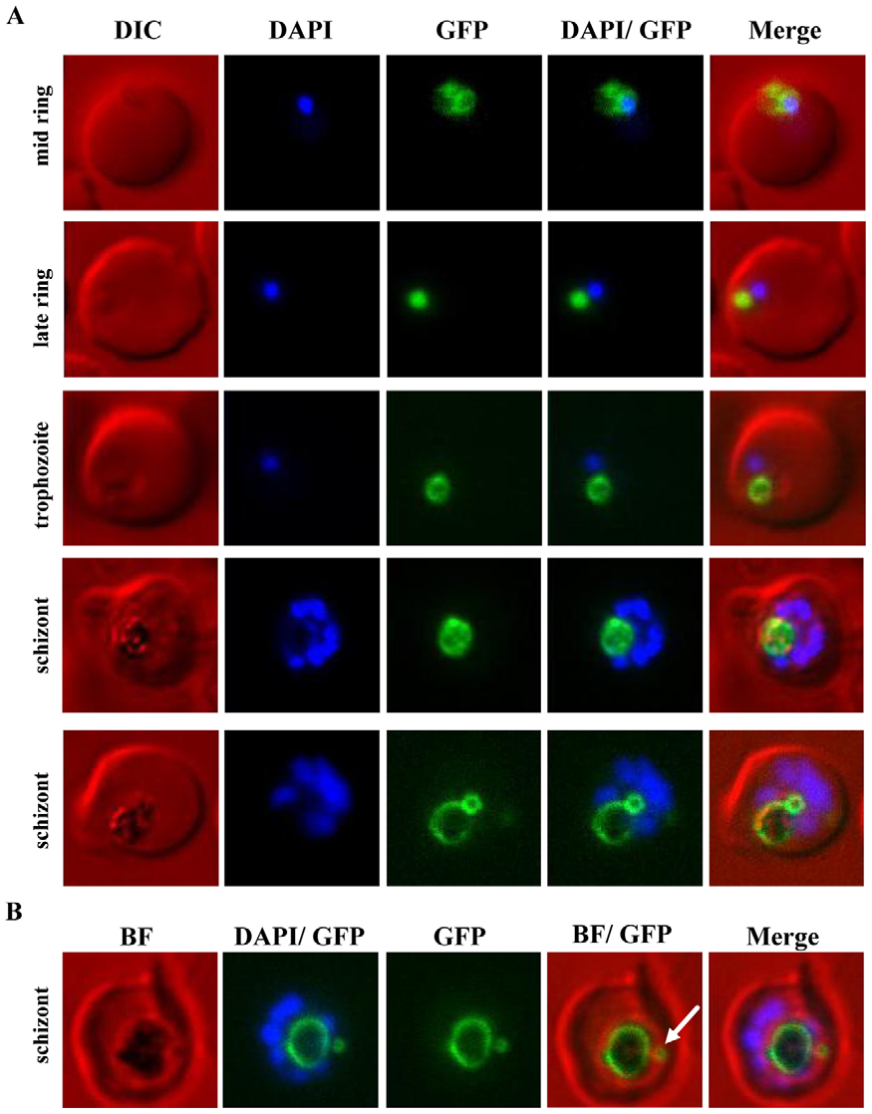
Analysis of the *de novo* biogenesis and development of the FV in live cells using PfCRT-GFP as a marker of the FV membrane. PfCRT was over-expressed as a GFP-fusion protein using an ATc-inducible expression system. (**A**) Live DAPI-stained cells were imaged. The earliest detectable and distinct localisation of PfCRT-GFP was observed in mid ring stage parasites, co-localising with a round spherical shape in proximity to the DAPI-stained nucleus. The characteristic dark haemozoin crystal was not yet visible in these parasites. In addition to FV labelling, some fluorescence was detectable at the ER. PfCRT-GFP labelling of the FV membrane was observed throughout the whole intra-erythrocytic life cycle. In accordance with an increase of the haemozoin crystal, the ring-like labelling of the FV membrane expanded as the parasite grew. An unexpected second PfCRT-GFP-labelled sphere was observed in schizont stage parasites. In some parasites this additional structure was associated/attached to the FV membrane (lower schizont panel). (**B**) An overlay with the corresponding brightfield (BF) image shows that in some parasites the additional PfCRT-GFP enclosed compartment (white arrow) is separate from the FV and surrounds a dark structure, possibly haemozoin.

The focus of previous studies of PfCRT were either its localisation or trafficking to the FV membrane [22], [33] and these studies focused on trophozoite parasites because the FV was apparent during this life cycle stage and allowed co-localisation with the haemozoin crystal. We therefore wished to extend these studies by using the PfCRT-GFP fusion as a marker of FV development throughout the asexual intra-erythrocytic life cycle. The first expression of GFP-tagged PfCRT^S^ in induced parasites was detected as a juxta-nuclear dot-like structure in mid to late rings (Figure 3A). In mid ring parasites a dual localisation between the nascent FV and the ER was frequently observed, indicated as a juxta-nuclear puncta structure as well as a fine ring around the nucleus. In late ring stage parasites, the small sphere of fluorescence co-localises with a round spherical body that is visible by light microscopy, yet at this stage still lacks any detectable refractile or dark haemozoin crystal. This indicates that a discrete pre-FV compartment, bearing PfCRT, forms prior to the appearance of haemozoin, which is normally used as a morphological character to define commencement of the trophozoite stage (Figure 3A).

This stage was followed by enlargement of the PfCRT-delineated vacuole, then continued expansion of both the haemozoin crystal and the vacuolar membrane. In mature schizont stages, a second PfCRT^S^-GFP-labelled structure was observed that enlarged into a small ring structure that was either attached to the FV membrane or separate from the FV membrane (Figure 3A, B). This second ring structure, if not adjacent to the FV membrane, had no association with the FV whatsoever and seemed to be an independent organellar compartment itself. Haemozoin crystal was also apparent within these secondary FV-like compartments (Figure 3B). In summary, live cell microscopy of PfCRT-GFP parasites reveals the presence of a pre-FV compartment prior to detection of haemozoin crystal. This compartment may originate from the parasite periphery, but is also consistent with previously-described dynamic processes during late stage parasites that result in a second FV compartment originating from the FV [7], [56].

### Analysis of the targeting processes of GFP-tagged PfCRT^S^

**Figure 4:**
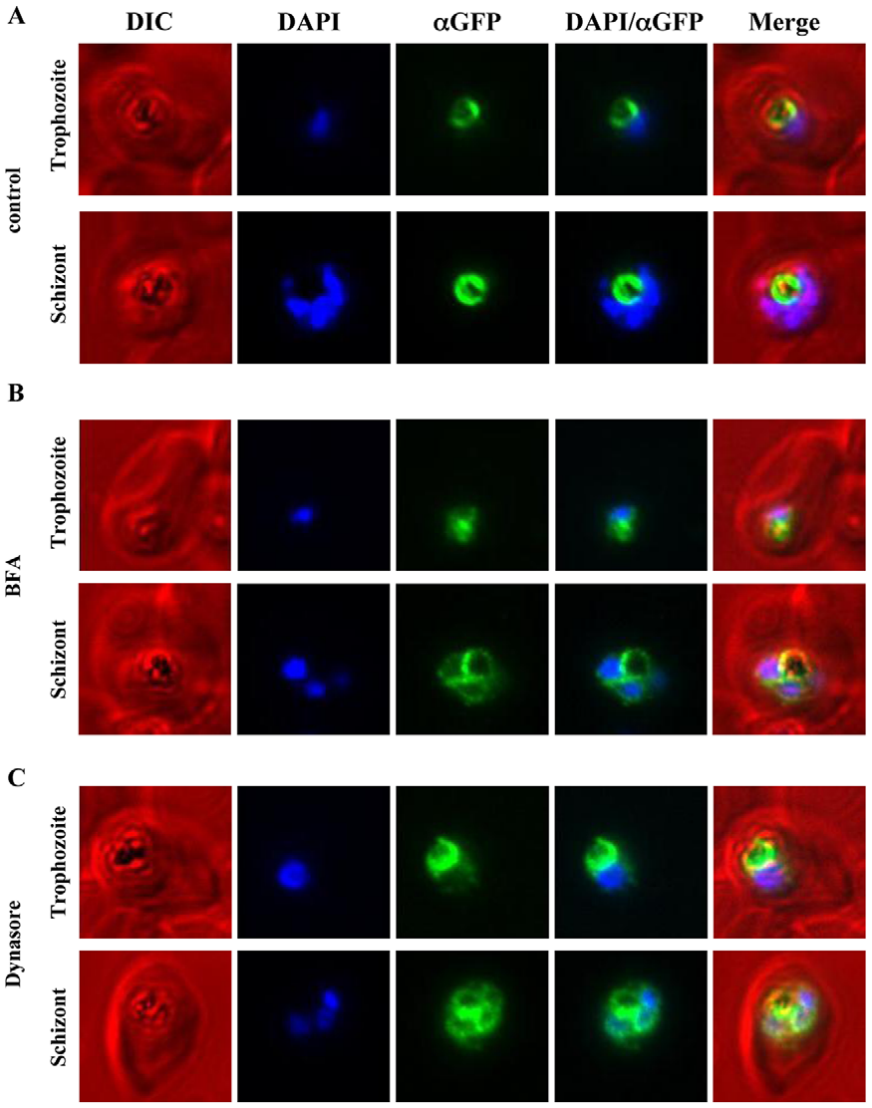
Immunofluorescence microscopy of PfCRT-GFP over-expressing parasites treated with either Brefeldin A or Dynasore. PfCRT was over-expressed as a GFP-fusion protein using an ATet-inducible expression system and treated with either BFA (5 µg/mL) for 3 h or Dynasore (40 µM/mL) for 2 h. As a control a second parasite population was treated with an equivalent volume of carrier alone (ethanol and DMSO, respectively). Following the BFA treatment, immunofluorescence microscopy was performed on fixed cells with mouse anti-GFP, Alexa-594 goat anti-mouse IgG and DAPI. Representative parasites in trophozoite and schizont stage are shown for control and treated parasites. (**A**) The control parasites show the restricted FV localisation of PfCRT-GFP (false-coloured in green). (**B**) BFA treated parasites show an accumulation of fluorescence around the DAPI-stained nuclei, consistent with ER localisation in addition to the FV localisation (Figure 1). (**C**) Treatment of parasites with Dynasore resulted in an accumulation of fluorescence around the DAPI-stained nuclei in addition to the FV membrane localisation, similar to the observed effect of BFA treatment (**B**).

Inducibly expressed GFP-tagged PfCRT^S^ was used to investigate the trafficking route of PfCRT to the FV membrane. The treatment of parasites over-expressing PfCRT^S^-GFP with BFA was performed in order to confirm the use of the inducible system for trafficking studies using GFP-tagged PfCRT^S^ [33]. BFA treatment of mainly trophozoite and schizont stage parasites was performed as previously described for 3 h with 5 µg/mL BFA [33]. Whereas the control population treated only with the equivalent volume of ethanol, showed a restricted FV membrane localisation (Figure 4A), the BFA-treated parasites showed a marked increase in fluorescence around the nucleus, corresponding to ER localisation (Figure 4B). As with the untreated parasites, there was no accumulation or labelling of PfCRT^S^ molecules at either the PPM or a Golgi-like compartment. These results were consistent with a previous study dissecting the targeting processes of PfCRT [33].

To investigate a role for dynamin-mediated vesicle formation in the trafficking of PfCRT^S^, we treated GFP-tagged PfCRT^S^ expressing parasites with the dynamin GTPase inhibitor Dynasore. Previous studies localised PfDYN1(PF3D7_1145400) to the ER and FV, whereas PfDYN2 (PF3D7_1037500) was localised to the ER and Golgi compartment [57], [58] and Dynasore treatment was reported to disrupt the integrity of the FV, producing a fragmented organelle [50]. We treated malarial parasites expressing GFP-tagged PfCRT^S^ for 2 h with a concentration of 40 µM, a concentration reported to reduce GTPase activity of both dynamin-like enzymes by approximately 50%, but which enabled the parasites to develop to later stages [50], whereas a concentration at 80 µM completely inhibited the development of the parasites. Analysis of parasites cultured longer than 2 h in the presence of Dynasore did not show any clear differences in morphology and labelling pattern compared to the 2 h treatment (data not shown). Fluorescence in parasites treated with Dynasore was somewhat lower, indicative of some toxicity, as seen with BFA. Immunofluorescence showed that while there was GFP at the FV membrane, there was also an increased accumulation of PfCRT^S^-GFP around the nucleus in the treated parasites, corresponding to ER localisation (Figure 4C). Some dynasore-treated parasites showed a pattern of PfCRT^S^ that did not perfectly co-localise with the FV but which formed a long string-form associated with the exterior of the FV without embracing the whole FV as in the untreated parasites. These data indicate that the trafficking of PfCRT is via the ER and not only involves BFA-sensitive processes, as previously shown [33], but also the action of dynamin-like proteins.

### Phenotypic analysis of malarial parasites over-expressing PfCRT^S^ and PfCRT^R^

Parasites inducibly over-expressing GFP-fusions of PfCRT^S^ and PfCRT^R^ were used to investigate differences in sensitivity to CQ. These parasites each possessed two separate PfCRT proteins – the unmodified genomic copy of PfCRT^S^ in the 3D7 line, as well as the GFP-fusion of either PfCRT^S^ (from 3D7) or PfCRT^R^ (from 7G8). Because episomes are poorly segregated in *P. falciparum*, some parasites in any generation of a transfected line do not express the desired product. In addition, the inducible system is imperfect, and only a small number of parasites strongly express the induced protein, as found in previous implementations of this system [44]. Moreover, expression of the PfCRT-GFP fusion became considerably weaker several cycles after induction, precluding longer-term experiments, or establishment of parasites stably expressing the fusion protein. The reason for this is unclear, but may relate to toxicity of longer expression of the PfCRT-GFP fusions. These variables meant that it was important to carefully measure the number of parasites expressing the PfCRT-GFP fusion in each line during the drug sensitivity assays, and to perform the drug sensitivity assays as soon as possible after induction. After fusion protein induction by removal of the transcription inhibitor ATc from sorbitol-synchronised ring stages, parasites were allowed to over-express PfCRT^S^-GFP or PfCRT^R^-GFP until the next ring stage after synchronisation, at which point the CQ drug assay was commenced. To assess consistency of PfCRT-GFP fusion expression, fluorescence (of induced parasites) was monitored and the percentage of GFP-expressing parasites determined in separate plates a further 12 hours later. On average 7.7±0.4% and 8.3±0.8% of parasites were fluorescent in the PfCRT^S^-GFP and PfCRT^R^-GFP lines, respectively. This result confirmed the consistency of the inducible expression system and additionally demonstrated comparable proportions of over-expressing parasites in the two independent populations after ATc removal.

Parasite sensitivity to CQ was followed using a SYBR-Green assay of parasite growth over 48 hours, with the WR99210 selective agent maintained in transfected parasites. From a total of five independent experiments, each with three internal replicates, the IC_50_ value for 3D7 wild-type parasites using this experimental method was on average 18.9±3.8 nM, and W2mef parasites had an IC_50_ for chloroquine of 295.3±21.0. (Table 1) These IC_50_ values are consistent with 48 hour IC_50_s previously reported for these strains [59], [60]. Parasites transfected with the PfCRT-GFP plasmids but with transgene expression repressed by maintenance on ATc showed no significant difference in the IC_50_ compared to 3D7 (t-test p>0.05). No significant difference in chloroquine sensitivity was observed between these parasites and parasites expressing either PfCRT^R^-GFP or PfCRT^S^-GFP (t-test p>0.05). Verapamil, a chloroquine-resistance reversing agent, restored much of the CQ sensitivity of the CQ-resistant W2mef, but had no discernible effect on the CQ sensitivity of the PfCRT^R^-GFP or PfCRT^S^-GFP expressing lines (Table 1) (t-test p>0.05). We conclude, therefore, that these fusion proteins either have no impact on chloroquine resistance, or a sufficiently mild effect that our drug-response assay is unable to detect it.

**Table 1:** IC_50_ of different *P. falciparum* transgenic lines and treatments after 48 hours chloroquine treatment.

Strain/treatment	IC_50_±SD
3D7/PfCRT^R^GFP uninduced	19.9±3.3
3D7/PfCRT^S^GFP uninduced	19.2±3.6
3D7 PfCRT^R^GFP induced	20.9±5.5
3D7 PfCRT^S^GFP induced	19.6±3.5
3D7 PfCRT^R^GFP induced + verapamil	18.1±1.7
3D7 PfCRT^S^GFP induced + verapamil	17.6±1.7
3D7wt	18.9±3.8
3D7wt + verapamil	18.0±0.4
W2MEF	295.3±21.0
W2MEF + verapamil	56.6±0.7

## Discussion

The food vacuole membrane protein PfCRT is a major modulator of chloroquine resistance [22]. The molecular basis of chloroquine resistance is still debated, but a likely mechanism is that mutations in PfCRT modify this transporter or channel so that it allows chloroquine to leak out of the FV. PfCRT from chloroquine resistant parasites bear mutations including an apparently crucial substitution of the lysine at position 76 [61], [62], [63]. Mutant, but not wild type PfCRT has been shown to transport chloroquine in *Dictyostelium* and *Xenopus ooocyte* models [30], [64]. Here we have studied PfCRT from resistant and sensitive parasites in *P. falciparum* by generating parasite lines with ATc-regulatable overexpression of these full-length PfCRT proteins fused to GFP. These parasites were used to address three questions; 1) Do mutations in the transmembrane domain of PfCRT alter its trafficking characteristics and thereby influence chloroquine sensitivity; 2) How does a model FV membrane protein traffic to the FV? and 3) Is over-expression of PfCRT from a resistant line sufficient to bestow CQ resistance in the genetic and epigenetic background of a CQ-sensitive parasite? The tools we developed proved to be insufficient to conclusively address the last question, but produced important data addressing the other two questions.

### Is the subcellular localisation of PfCRT^S^ identical to PfCRT^R^ at the FV membrane?

Experiments in other eukaryotic systems have increasingly made clear the important role of TMDs in positioning a protein within the cell. For proteins in the endomembrane system, the composition and length of the transmembrane domains, particularly the initial membrane-spanning domain, can partially determine their subcellular localisation [34], [35], [36], [42], [55], [65]. Changes that result in mis-targeting include mutations that alter the length of a transmembrane domain [35], or which add or remove a polar face to a transmembrane domain (eg [42]) (Figure 2C). Specific transmembrane domains have also been shown to be instrumental in trafficking in *Plasmodium falciparum* [66], [67]. The major resistance determining mutation in PfCRT is at the end of the first predicted transmembrane domain [22], and multiple additional mutations are found in other transmembrane domains of PfCRT that modulate resistance to chloroquine and other quinolone drugs [62]. Bioinformatic interrogation of these mutations suggest that they may alter the position and length of the initial transmembrane domain (Figure 2). We therefore tested whether changes in these transmembrane domains may modulate resistance to *Plasmodium* by changing the subcellular localisation of PfCRT – potentially altering subcellular distribution of chloroquine by changing relative amounts of the transporter on the food vacuole, plasma membrane or ER/Golgi. Our examination of identically-prepared GFP fusions of resistant and sensitive forms of PfCRT conclusively falsifies this hypothesis – there was no difference in abundance or localisation between the two forms, and both were exclusively intracellularly localised, with no detectable fraction at the plasma membrane.

A much more likely alternative is that the changes in charge and potential orientation changes in the membrane spanning portion of the protein brought about by changed TMD lengths result in an altered transport capacity of PfCRT. Cooper and colleagues [62], and Martin and Kirk, have convincingly described how the diminished positive charge at this limit of the PfCRT TMDs polar face [68] might change the transporter's affinity for protonated CQ^2+^. The increased ability of the PfCRT^R^ to transport chloroquine in heterologous systems is strongly supportive of this model [30], [64].

### How does PfCRT traffic to the FV?

We used the inducible PfCRT-GFP fusions to investigate the trafficking route of induced protein to the FV membrane. Kuhn and colleagues have previously demonstrated that PfCRT traffic to the FV membrane is via the ER/Golgi using a combination of genetic deletion experiments and treatment of parasites with the fungal metabolite Brefeldin A (BFA) [33]. BFA interferes with early targeting events between ER and Golgi level by inhibiting the function of guanine nucleotide-exchange (GEP) proteins that regulate the activation of ADP-ribosylation factor (Arf) proteins [69], [70]. Arf proteins are involved in a wide range of functions, including regulation of ER and Golgi function and morphology, ER-to-Golgi transport, and recruitment of COP and adaptor protein complex proteins (reviewed in [71]). We also examined the BFA sensitivity of the inducibly expressed PfCRT-GFP fusion, and found that BFA produced a defect in traffic from the ER/Golgi to the FV membrane, consistent with a trafficking route via the ER/Golgi (Figure 4). One means of trafficking vesicles from the ER and Golgi is through dynamin and dynamin-like GTPase-mediated vesicle budding [72], [73], [74], [75]. *Plasmodium* has several dynamin-like proteins, some likely involved in organelle division and endocytosis, and others potentially involved in vesicle budding [58], [76], [77]. One of these dynamin homologues – PfDYN1 localised to multiple cellular destinations including the FV and the ER [58], while another, PfDYN2 was reported to localise to the ER, Golgi compartment and the relict plastid [57]. Roles for dynamin-like GTPases in *Plasmodium* have been interrogated using Dynasore, a non-competitive inhibitor that acts on the dynamin GTPase domain [78]. Dynasore has been reported to produce a dramatic effect on *Plasmodium* endocytosis and haemoglobin uptake, as well disrupting leading to profound fragmentation of the FV [50].

We addressed whether dynamin-like GTPases are involved in the targeting of PfCRT to the FV by Dynasore-treating parasites that over-expressed PfCRT^S^-GFP. In contrast to a previous study [50], we saw no widespread morphological defects of the FV, assayed by live cell imaging of parasite with the FV membrane highlighted by PfCRT^S^-GFP labelling (figure 4C). In our experiments the FV was intact and not fragmented. We did however see some disruption of PfCRT^S^-GFP targeting, with an increase of fluorescence around the nuclei, corresponding to ER localisation (figure 4C). This suggests that dynamin-like proteins may be involved in the trafficking of proteins from the ER/Golgi to the FV, potentially through its action in completing budding of vesicles. These data also independently support the model of an ER-mediated trafficking mechanism for FV membrane proteins.

### How does the food vacuole develop?

The FV does not persist throughout the intra-erythrocytic life stages and hence depends on *de novo* biogenesis following the invasion of each new erythrocyte. Current models that try to describe how the FV *de novo* biogenesis occurs [5], [6], [7] and how this endosomal/lysosomal-like organelle develops over the intra-erythrocytic life cycle [79], [80] are based on observations from various microscopic techniques. The availability here of a fluorescent marker of the FV membrane meant that we were able to identify mid to late ring stage parasites – even before haemozoin crystals are present – as the earliest parasites with a defined FV compartment. Abu Bakar and colleagues showed the existence of various small pre-FV compartments that fuse together to form a central FV compartment [7]. Light microscopy likely provides insufficient resolution for us to be able to identify whether the earliest PfCRT labelling delineates one small compartment, or multiple small vesicles aggregating. Experiments using fluorescent tracers of the erythrocyte cytoplasm [7] could elucidate whether these earliest PfCRT-bound compartments already contain haemoglobin.

The other interesting finding respecting FV cell biology was the revelation of a second dot-like structure or ring structure beside the FV ring localisation in later schizont stage parasites. Several studies of the FV do indeed show neighbouring compartments that might contain haemozoin, but it was unclear if those compartments were protrusions of the FV whose connections were invisible in 2 dimensions [56], [61]. Instead, these smaller PfCRT-GFP compartments share more similarities with long-lived extra-FV compartments described by Abu Bakar and colleagues [7]. It is unclear if these secondary compartments derive from parcels of haemoglobin from the cytostomal invagination that have already recruited PfCRT but have fail to fuse with the existing FV membrane, or if they result from the pinching-off of a part of the FV resulting in a second FV-like compartment.

Another possibility for the identity of these structures are neutral lipid bodies, which are superficially similar in appearance. Neutral lipid bodies can be visualised with the hydrophobic probe Nile Red and they have been previously linked to haemozoin formation [81], [82]. In *Plasmodium* they have been found within the FV lumen and in schizont stage parasites in the cytosol as well. However, an association between PfCRT and neutral lipid bodies was not detected in a western blot analysis of neutral lipid bodies [81]. Furthermore, in contrast to the multiple neutral lipid bodies seen in individual schizonts [82] we saw only a single additional PfCRT bound compartment in each cell. The substantial emission spectrum overlap between Nile Red and GFP prevented colocalisation experiments with PfCRT-GFP using available imaging equipment.

### Chloroquine sensitivity in PfCRT-GFP over-expressing parasites

Despite the clear causal relationship between mutations in PfCRT and chloroquine resistance, the role of additional genetic and epigenetic factors in the acquisition of chloroquine remains unclear. This is partly because other lines expressing transgenic PfCRT [22] or with PfCRT allelic exchanges [63], [83] have had weeks of growth at least to compensate for any negative effects of the mutant PfCRT [32]. We attempted to study whether sudden expression of mutant, chloroquine resistant PfCRT in a sensitive background was sufficient to bestow chloroquine resistance by using the repressible ATc expression system. However, the IC_50_ values obtained for CQ sensitive 3D7 parasites over-expressing either PfCRT^S^-GFP or PfCRT^R^-GFP showed no significant difference. Several factors make these results difficult to interpret - the first is that only a small percentage (only ∼8–10%) of cells expressed the fusion proteins after induction in either cell lines. This effect has been seen before for ATc-regulatable systems in apicomplexan parasites [44], and in our system meant that a large number of the parasites being assayed were in fact parental population, and any phenotypic signal emanating from the induced parasites would have been weakened by the background signal from the non-expressing parasites. Nevertheless, the gap in drug sensitivity between strongly drug resistant and sensitive lines is sufficient that substantial differences should still have been apparent as a changed IC_50_ even in a mixed population. Another possible confounding factor is that the GFP fusion to PfCRT may prevent these from functioning as transporters, or may inhibit their interaction with chloroquine. We had initially intended to study inducibly over-expressed PfCRT forms without the GFP fusion to investigate this effect. However, the very low levels of parasites expressing after induction) meant such an experiment would have been unreliable without a marker for the PfCRT overexpression. Some PfCRT antibodies are available, but none were reliably specific in our hands.

FACS based sorting of fluorescent parasites to purify populations of homogeneously expressing cells may represent another potential experimental approach but the low number of cells possible to recover in such a sorting experiment may be inappropriate for use in a drug sensitivity assay. Development of a superior inducible system in *P. falciparum* may also address these issues. A conditional protein stabilisation system has been used to excellent effect to address several questions of *Plasmodium* biology, but the applicability of this system to all subcellular destinations is uncertain [84], [85].

### Summary

PfCRT as a marker protein of the FV membrane has proven to be an important tool to study the *de novo* biogenesis and development of the FV in live *Plasmodium* parasites. However, the exact process underlying the *de novo* biogenesis of the FV and the mechanism by which haemoglobin is transferred to the FV remain important questions to address. So too does the question of how important compensatory mechanisms are for allowing PfCRT mutations to mediate chloroquine resistance. The latter question would be particularly facilitated by a superior inducible expression system that enables robust interrogation of conditionally expressed PfCRT.
